# Poster Session I- Poster of Distinction - A46 IMPROVING INTER-OBSERVER AGREEMENT IN ENDOSCOPIC FINDINGS WITH STANDARDIZED TERMINOLOGY

**DOI:** 10.1093/jcag/gwaf042.046

**Published:** 2026-02-13

**Authors:** Y Song, S Samnani, V J Arroyo, R Gaikar, A Azad, D Armstrong

**Affiliations:** McMaster University, Hamilton, ON, Canada; McMaster University, Hamilton, ON, Canada; McMaster University, Hamilton, ON, Canada; AI VALI, Toronto, ON, Canada; AI VALI, Toronto, ON, Canada; McMaster University, Hamilton, ON, Canada

## Abstract

**Background:**

A shared, standardized vocabulary for endoscopic diagnoses is essential for accurate reporting, clear communication and machine learning (ML). Heterogeneity in report language can lead to inconsistent labeling of findings, inter-observer disagreement, variation in patient management and suboptimal ML. Advances in digital imaging create opportunities to standardize imaging descriptors across providers and evaluate whether harmonized terminology reduces disagreement.

**Aims:**

To assess whether standardizing diagnostic terminology decreases inter-observer disagreement for endoscopic findings.

**Methods:**

Three gastroenterology (GI) residents independently assigned diagnoses to 150 endoscopic images (esophagogastroduodenoscopy, colonoscopy). An expert faculty GI at an academic center, blinded to resident assessments, reviewed the same images to establish reference diagnoses. Inter-observer disagreement between residents and the expert was calculated to generate a pre-standardization score. The residents then collaborated with the expert to develop a standardized terminology set, which was used to relabel all 150 images, and inter-observer disagreement was recalculated to generate post-standardization score. Fleiss Kappa score was then calculated to determine disagreement between four endoscopists before and after standardization.

**Results:**

Overall, disagreement scores were lower for landmark recognition in esophagogastroduodenoscopy (EGD) than in colonoscopy, whereas disagreement in lesion diagnoses was lower in colonoscopy than in EGD. The largest increases in Fleiss’ kappa were observed after standardizing landmark-recognition terminology for EGDs. Details of disagreement and kappa metrics are provided in Table 1.

**Conclusions:**

Standardizing terminology in endoscopy reports improves inter-observer agreement and reduces interpretive discrepancies. Agreement on major anatomic landmarks exceeded agreement on diagnostic labels for EGDs in particular, highlighting substantial variability in descriptors for endoscopic findings. Fleiss’ kappa increased most for landmark recognition in EGD, indicating that harmonized nomenclature can markedly enhance documentation consistency in this domain. Larger, multi-center studies with a broader spectrum of lesions are warranted to validate these results and to quantify the impact of report vernacular on clinical decision-making and patient outcomes.

**Funding Agencies:**

None

**A46 Table 1:** Pre & post standardization disagreement scores (%) vs expert GI: 150 images

Category	Procedure Type	Resident 1		Resident 2		Resident 3		Fleiss Kappa Score	
		Pre	Post	Pre	Post	Pre	Post	Pre	Post
Landmark recognition	EGD	27.0	15.1	28.4	17.8	18.9	6.9	0.76	0.84
	Colonoscopy	37.3	37.3	50.7	49.3	41.3	41.3	0.44	0.46
	Overall score	37.2	26.2	39.5	33.6	30.1	24.2	0.60	0.65
Diagnosis	EGD	41.9	34.3	46.0	39.7	40.5	32.9	0.40	0.41
	Colonoscopy	17.3	13.3	16	12.0	14.7	12	0.79	0.79
	Overall score	29.3	23.5	30.7	25.5	27.3	22.2	0.60	0.60

